# A (further) test of spontaneous serial refreshing in verbal and spatial working memory

**DOI:** 10.3758/s13414-022-02624-x

**Published:** 2022-12-01

**Authors:** Evie Vergauwe, Naomi Langerock

**Affiliations:** Faculté de Psychologie et des Sciences de l’Education, Université de Genève, 40 bd du Pont d’Arve, 1211 Genève 4, Switzerland

**Keywords:** Working memory, Attention

## Abstract

Working memory is the cognitive system that keeps a limited amount of information temporarily accessible for ongoing cognition. One proposed mechanism to keep information active in working memory is *refreshing*. Refreshing is assumed to operate serially, reactivating memory items one by one by bringing them into the focus of attention during retention. We report two experiments in which we examine evidence for the spontaneous occurrence of serial refreshing in verbal working memory (Experiment 1, using letters as memoranda) and in visuospatial working memory (Experiment 2, using locations as memoranda). Participants had to remember series of red memory items, and black probes were presented between these memory items, with each probe to be judged present in or absent from the list presented so far, as quickly as possible (i.e., the probe-span task). Response times to the probes were used to examine whether the content of the focus of attention changed over time, as would be expected if serial refreshing occurs spontaneously during interitem pauses. Contrary to this hypothesis, our results indicate that the last-presented memory item remained in the focus of attention during the interitem pauses of the probe-span tasks. These findings confirm the boundary conditions of spontaneous serial refreshing that were previously observed for verbal working memory and extend them to visuospatial working memory. Implications for working memory maintenance are discussed.

**Supplementary Information:**

The online version contains supplementary material available at 10.3758/s13414-022-02624-x.

Working memory is the cognitive system that keeps a limited amount of information temporarily accessible for ongoing cognition. One major question in the field is how information is maintained in working memory, such that information can still guide behavior when it is no longer perceptually available. Several maintenance processes have been proposed to support working memory storage (see Oberauer, 2019, for a recent review). Here, we focus on one maintenance process in particular, that of *refreshing*. Refreshing has been proposed as a domain-general, attention-based maintenance process that reactivates to-be-remembered information by bringing the information into the focus of attention during retention (e.g., Barrouillet & Camos, 2012; Cowan, 1995; Higgins & Johnson, 2009; Vergauwe & Cowan, 2014). Originally proposed in the 1990s, as a basic process that sustains or prolongs temporary activation of already active information in memory within the multiple-entry modular memory system (MEM) framework (M. K. Johnson, 1992), refreshing has been introduced more explicitly as a maintenance process in the early 2000’s by the time-based resource-sharing (TBRS) model (see Barrouillet & Camos, 2021, for a recent review). Refreshing has received much attention over the last 15 years (e.g., Atkinson et al., 2022; Bartsch et al., 2018; Camos et al., 2019; Káldi & Babarczy, 2021; Lintz & Johnson, 2021; Oberauer & Souza, 2020; Vergauwe et al., 2019) and is now included as one of the key working memory maintenance processes in recent versions of several working memory models (e.g., Baddeley et al., 2021; Barrouillet & Camos, 2021; Cowan et al., 2021; Shimi & Scerif, 2017; Vandierendonck, 2021). Despite the growing body of research on refreshing, and the growing number of models adopting refreshing as a key maintenance process in working memory, it is currently still unclear how refreshing operates to support the maintenance of a set of elements in working memory.

The operation of refreshing is often described by contrasting it with the operation of verbal rehearsal (see Camos, 2017, for a recent overview). Whereas verbal rehearsal is assumed to rely on subvocal articulation of information, refreshing is assumed to rely on attention-based reactivation of information. Furthermore, whereas verbal rehearsal is a speech-related maintenance mechanism that can only be used to maintain verbal information, refreshing is an attention-based mechanism that can be used to maintain different types of information, including visuospatial information. Specifically, refreshing is assumed to operate by bringing working memory representations into the focus of central attention (e.g., Barrouillet & Camos, 2012; Cowan, 1995; Higgins & Johnson, 2009; Vergauwe & Cowan, 2014), and its use is therefore not limited to verbal information. Information in the focus of attention is assumed to be in a privileged state of heightened accessibility and thus bringing working memory representations into the focus of attention during retention is assumed to result in working memory representations becoming highly accessible again, regardless of their nature. This, in turn, is proposed to protect the information from forgetting, with better memory performance as a result.

An important similarity between verbal rehearsal and refreshing is that both are assumed to operate serially. Indeed, the most prominent hypothesis about the operation of refreshing proposes that refreshing operates serially, with the focus of attention cycling from one item to the next, thereby reactivating working memory representations sequentially (e.g., Barrouillet & Camos, 2012; Cowan, 2011; McCabe, 2008; Nee & Jonides, 2013; Vergauwe et al., 2014). In two recent studies, we have tested this hypothesis but did not find evidence for serial refreshing (Vergauwe et al., 2016, 2018). In these studies, we used the probe-span task in which short lists of to-be-recalled red letters were presented. The presentation of each to-be-recalled letter was followed by a black memory probe to be judged present in or absent from the list presented so far, as quickly as possible. We manipulated the preprobe delay—that is, the free time that separates the offset of the red memory item (or the mask, in some cases) from the onset of the black probe letter—and the idea was to infer the occurrence of serial refreshing from a specific pattern in response times that would change over time if participants use said time to refresh the to-be-recalled letters. In particular, in some conditions, the preprobe delay was very short (e.g., 100 ms), and we reasoned that, with very brief preprobe delays, there is no time for refreshing in between the last-presented memory item and the probe. As a result, with very brief preprobe delays, the last-presented letter remains in the focus of attention. Because the item in the focus of attention is assumed to be in a privileged state of heightened accessibility (e.g., Basak & Verhaeghen, 2011; Cowan, 1995; McElree, 2006; Nee & Jonides, 2008; Oberauer & Hein, 2012), we expected that responses to probes matching the last-presented letter would be speeded, relative to responses to probes matching any of the other to-be-remembered letters when very short preprobe delays were used (i.e., last-presented benefit). With longer preprobe delays, however, there is time to refresh the to-be-remembered letters before the probe is presented. As a result, with longer preprobe delays, if serial refreshing occurs spontaneously, the last-presented letter is replaced in the focus of attention. Because the focus of attention is assumed to rotate among the different to-be-remembered letters in working memory during serial refreshing, we no longer expected speeded responses to probes matching the final to-be-remembered letter when longer preprobe delays were used (i.e., abolishment of last-presented RT benefit). Against our expectations, across a total of six experiments, the last-presented RT benefit was never abolished (Vergauwe et al., 2016, 2018); responses remained the fastest for the last-presented memory item, at all preprobe delays. This indicates that the last-presented item was still in the focus of attention when the probe was presented and thus, that serial refreshing had not occurred during longer preprobe delays. The current study follows up on this unexpected pattern of results. In particular, we propose two alternative interpretations of the results of Vergauwe et al. (2016, 2018) and propose two experiments to test these alternatives.

According to the first alternative account, we did not find evidence for serial refreshing in the aforementioned studies (Vergauwe et al., 2016; Vergauwe et al., 2018), because the periods of free time that were provided between memory items and probes were too short for refreshing to occur. The longest preprobe delays that were used in these studies had a duration of 800 ms. Refreshing is typically assumed to operate rather quickly, at a speed of less than 100 ms per item (80 ms per item, proposed by Oberauer & Lewandowsky, 2011; 40–50 ms per item, proposed by Barrouillet & Camos, 2014; Vergauwe et al., 2014; Vergauwe & Cowan, 2014). Other studies suggested a slightly slower speed of 200 ms per item (Oberauer & Souza, 2020), which fits rather well with the observation of retro-cuing benefits using time windows of about 150–250 ms (e.g., Souza et al., 2016). Based on these estimates of the speed of refreshing, 800 ms should be enough to refresh a small number of items. However, it could be argued that some additional time is needed for the focus of attention to switch away from the last-presented memory item, thereby initiating refreshing. Indeed, some researchers have proposed that refreshing consists of two subcomponents, and that the first subcomponent, i.e., the initiation of refreshing, takes about 400 ms (M. R. Johnson et al., 2015). If we assume that the initiation of refreshing takes about 400 ms, then only some of the longer preprobe delay durations that have been used, would have been long enough for refreshing to take place (i.e., preprobe durations of 600 or 800 ms). Importantly, however, these longer preprobe delay durations were always randomly intermixed with much shorter preprobe delay durations in the previous studies (i.e., intermixed with preprobe durations as short as 100 or 200 ms). It is possible that the inclusion of very short preprobe delay durations—that is, preprobe durations that are shorter than the time it may take to initiate refreshing—generally discouraged participants to engage in refreshing in our previous studies. In line with this idea, Vergauwe et al. (2019) recently showed that whether or not participants use free time in between list items to refresh all list items presented up to that point depends on time-based parameters of the task context rather than on the actual time available for refreshing. In particular, it was found that periods of free time between list items were used differently depending on the task context, such that short periods of free time were used for refreshing when they were intermixed in a slower task context of longer preprobe durations but not when they were intermixed in a faster task context of shorter preprobe durations. To examine the possibility that a faster task context discouraged participants to engage in refreshing in previous experiments, the current Experiment 1 used a probe-span task in which series of four red letters had to be remembered, but using longer preprobe delays than in previous studies, thereby creating a slower task context. All preprobe delay durations were now considerably longer than the time it may take to initiate refreshing; preprobe delays of 800 ms, 1,100 ms, and 1,500 ms were used. To anticipate, we replicated our previous observations and observed very strong evidence for a last-presented benefit for all preprobe delays, suggesting that no serial refreshing occurred between the memory items and the probes, despite the use of a slower task context. This observation led to proposing a second alternative account.

According to the second alternative account, evidence for serial refreshing was found neither in the current Experiment 1, nor in the aforementioned studies (Vergauwe et al., 2016, 2018), because participants relied mainly on verbal rehearsal to maintain the memory items. Verbal rehearsal and refreshing are assumed to be independent (e.g., Camos et al., 2009), and it has been shown that participants can adaptively favor one maintenance mechanism over the other (Camos et al., 2011). As such, it is possible that no evidence for serial refreshing was found in the previous experiments, because participants were relying on verbal rehearsal rather than on refreshing to maintain the information. Indeed, in all studies up till here, the memoranda in the probe-span task were letters (i.e., verbal materials that can easily be rehearsed). In some of the previous experiments, as well as in the current Experiment 1, we attempted to minimize the use of rehearsal by using phonologically similar letters as memoranda, for which Camos et al. (2011) have shown that participants favor refreshing over rehearsal. However, given that verbal memory materials were used, we cannot exclude the possibility that participants were mainly using verbal rehearsal to maintain the memoranda in our previous experiments. To address this issue in another way, the current Experiment 2 employed a spatial version of the probe-span task. Participants were presented with series of four red to-be-remembered locations, each red location being followed by a probe. Like in Experiment 1, we manipulated the preprobe delay duration (150 ms or 1,200 ms). Importantly, to minimize verbal encoding, locations were presented in a random grid. Previous research has shown that visuospatial memory materials mainly depend on attentional maintenance processes, as opposed to verbal materials, which can also use verbal rehearsal (e.g., Morey, 2018; Vergauwe et al., 2014), and, accordingly, visuospatial memory performance has been shown to suffer much less from concurrent articulation of syllables, an effect typically assumed to reflect the use of verbal rehearsal (referred to as articulatory suppression; e.g., Alloway et al., 2010; Vandierendonck et al., 2004; Vecchi & Richardson, 2001). While replacing letters by randomized spatial locations does not allow ruling out *any* involvement of verbal rehearsal, it should result in a drastic reduction in the use of verbal rehearsal. Experiment 2 allows us to examine whether evidence for serial refreshing can be observed when we further minimize a potential role of verbal rehearsal, and the use of spatial stimuli also allows us to provide a first assessment of the evidence for serial refreshing in visuospatial working memory. The data for all experiments can be accessed through the Open Science Framework (https://osf.io/5w6zd/).

## Experiment 1

### Method

#### Participants

Forty-three undergraduate students at the University of Geneva participated and received partial course credit. All had normal or corrected-to-normal vision. In a first batch, 31 participants were tested. After that, testing continued until we reached 30 data sets after performance-based exclusions (see below). The sample size is based on similar studies using the probe-span task (e.g., Vergauwe et al., 2018).

#### Materials and procedure

**Fig. 1:**
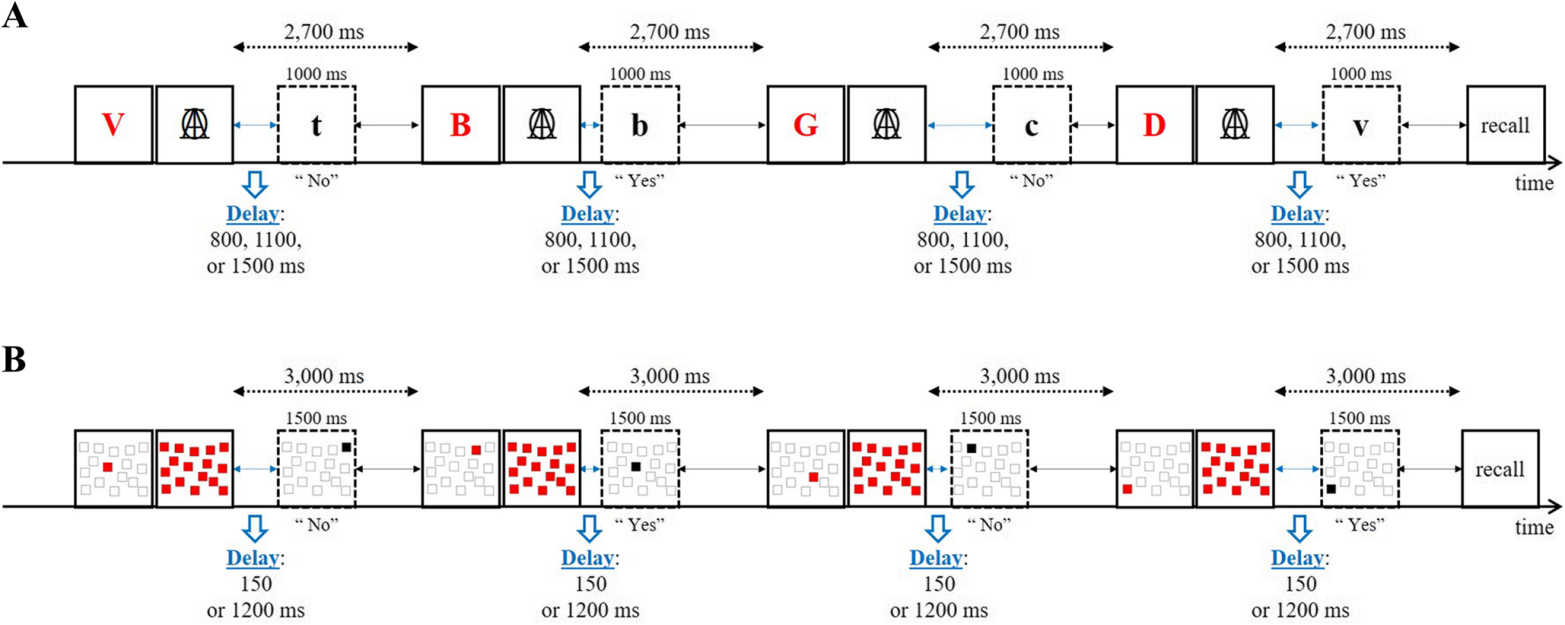
Illustration of a trial within the probe-span task used in Experiment 1 (**a**) and in Experiment 2 (**b**). Series of four red memory items were presented (and masked) for subsequent recall and black probe items were presented between the items to be remembered, with each probe to be judged present in or absent from the list presented so far. At the end of the series, participants recall the four memory items in order of appearance. The delay before the probe was manipulated (800, 1,100, or 1,500 ms in Experiment 1; 150 or 1,200 ms in Experiment 2). (Color figure online)

The probe-span task was administered using E-Prime 2 software (Psychology Software Tools). Participants were asked to watch carefully and memorize series of four red letters presented sequentially on screen (see Fig. 1a). Participants were asked to watch carefully and memorize series of four red letters presented sequentially on screen. Like in Experiment 4 of Vergauwe et al. (2016) and Experiment 2 of Vergauwe et al. (2018), a restricted pool of seven phonologically similar consonants (in French) was used as stimuli (B, C, D, G, P, T, and V), and each red letter was followed by a mask consisting of three superimposed black letters (*A*, *I*, and *O*), presented in uppercase 32-point Courier New font. These letters were used approximately equally often, and no letter was repeated within a series. Red letters were presented at the center of the screen in 48-point Courier New font, in uppercase. Stimuli were presented on a standard CRT monitor, and participants sat at a comfortable distance from the screen.

Each series began by a fixation cross, centrally displayed on screen for 750 ms, and followed by the first red letter. Red letters were presented for 500 ms. At the end of each series, an empty rectangle appeared on screen, prompting the participant to recall the four red letters of the series in order of appearance by typing them on the keyboard. Participants were encouraged to fill in unknown letters with a guess. All entered letters appeared in the box in uppercase, from left to right. Participants pressed enter to end the recall response and initiated the next series by pressing a button on the button box after recall.

After each to-be-remembered red letter, one black letter (probe) was presented in lowercase in the center of the screen in 24-point Courier New font. Participants were instructed to decide whether this black letter corresponded to one of the red letters they were to maintain on the current trial or not. This judgment was made by pressing the rightmost button of the button box when the black letter corresponded to one of the red letters in memory and pressing the leftmost button when the black letter did not correspond to one of the red letters in memory.

The preprobe delay variable was manipulated within-subjects. Regardless of the preprobe delay condition, the interval between two red letters was kept constant at 2,750 ms (50-ms mask, followed by a 2,700-ms window during which the probe was presented). Depending on the experimental condition defined by preprobe delay, the delay between the offset of the mask and the onset of the black probe letter was different (800, 1,100 or 1,500 ms). Preprobe delay varied within a trial and thus, probe onset was unpredictable. Probe letters were always presented for 1,000 ms. The remaining delay between the offset of the black letter and the onset of the next red letter differed as a function of preprobe delay (900, 600, or 200 ms, respectively).

The experiment consisted of 144 trials and, for each trial and each participant, black letters were sampled randomly from a pool of potential probes in such a way that the likelihood of receiving a positive probe was 50% at each probe position. Thus, each trial could have 0 to 4 positive probes. For each probe position, the pool of possible probes consisted of all the letters presented in the series so far plus a random new letter for that series. Across the entire experiment, and for each of the four probes, the black letter corresponded in half of the trials to one of the to-be-remembered red letters, and each red letter presented up to that point in the trial had equal chances of being used as target-present probe. Importantly, in each of the four pools of potential probes (i.e., one pool per probe position), every different probe type was associated equally often with each of the possible levels of preprobe delay.

Before the experimental trials, participants received instructions that included a visualization of a trial together with the experimenter. This was followed by five practice trials. Throughout the experiment, participants were asked to respond as fast as possible to the probes, without making errors, while maintaining the four red letters. They were not informed on the varying delays. Responses in the processing task were collected by button presses on a serial response box. Recall performance was scored by counting the number of letters that were correctly recalled with respect to serial order within each series (max = 4). Next, an average across all series was calculated per participant.

### Results and discussion

#### Performance-based exclusions

We applied the same performance-based exclusions as in Vergauwe et al. (2016, 2018), to keep things consistent across studies. First, we planned to discard the data of participants whose average recall score was, on average, less than one letter out of four. This did not lead to any data exclusion. Next, we excluded the data of participants who performed poorly on the probe task. This was done to ascertain that participants paid sufficient attention to the probe task. As in Vergauwe et al. (2016, 2018), poor performance was operationalized as a rate of correct responses below 55%. Two participants did not reach this criterion. Finally, we verified participants’ precise compliance with the instructions in the probe task. Therefore, like in Vergauwe et al. (2016, 2018), we calculated the rate of correct responses to “not-last” probes (i.e., target-present probes that show any-but-the-last-presented red letter of a series) and excluded the data of participants who scored below 55% on these not-last probes (11 participants). This last criterion is used because it is important that participants consider all of the red letters when judging the probe and not simply the last-presented red letter. These exclusions resulted in a final sample of 30. These participants correctly recalled several memory items at the end of the series (*M* = 3.43, *SD* = .41) and had high accuracy on the probes (*M* = .89, *SD* = .04).

#### Last-presented benefit

**Fig. 2:**
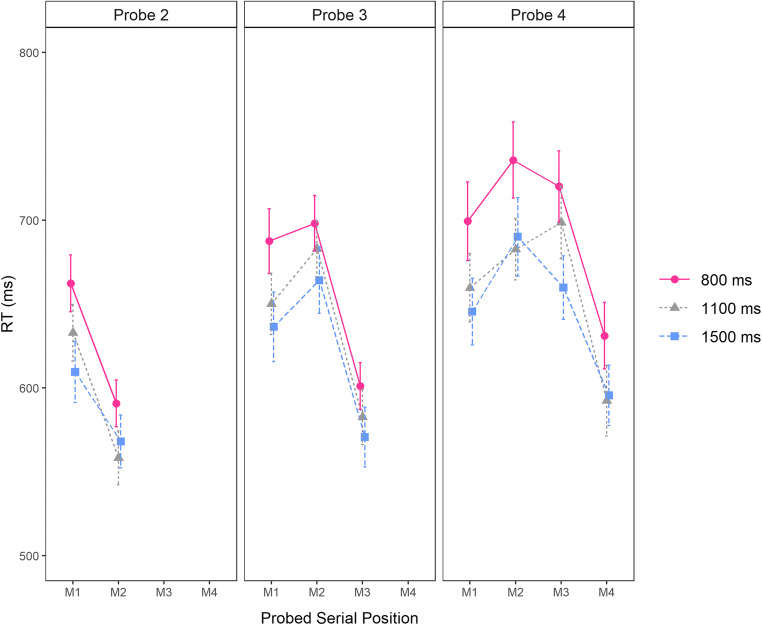
Mean probe response RT in ms observed in Experiment 1, as a function of the serial position of the matching memory item (probed serial position; on the *x*-axis) and probe position (Probe 2, Probe 3, or Probe 4 in the left, middle, and right panels, respectively). The delay following the probe appears as the graph parameter 800, 1,100, or 1,500 ms). Error bars show standard errors of the mean

Serial position curves for the RTs of correct responses collected at Probe Position 2, 3, and 4, (i.e., the probe letters following Memory Items 2, 3, and 4, respectively) are shown in Fig. 2. In line with our previous findings in verbal working memory, RTs were affected by the serial position of the matching memory item and became faster over time (i.e. with longer preprobe delay durations). Importantly, however, and still in line with our previous observations in verbal working memory (Vergauwe et al., 2016, 2018), there is no drastic change in the serial position curves over time. Instead, at all probe positions and for all preprobe delay durations, the last-presented item was the fastest responded to.

We tested the serial refreshing hypothesis by examining the evidence for or against a last-presented benefit at each preprobe delay duration. Therefore, for each preprobe delay duration (i.e., 800, 1,100, or 1,500 ms), we compared RTs to target-present probes matching the last-presented memory item to RTs to target-present probes that did not match the last-presented memory item (i.e., that matched another red letter of the current series). For each preprobe delay duration, and for each probe position, a separate one-sided Bayesian *t* test was run (i.e., nine *t* tests in total), testing whether the RTs to probes matching the last-presented memory item were faster than RTs to other target-present probes (i.e., a last-presented benefit in RT; see Vergauwe & Langerock, 2017).

Table 1 presents the results of these analyses. If spontaneous serial refreshing occurs during the delay before the probe, the last-presented item is assumed to be replaced in the focus of attention by another memory item and, as a result, the last-presented benefit should be abolished. In sharp contrast to this prediction, the evidence for a last-presented benefit was extremely strong at all preprobe delay durations, with Bayes factors ranging between 204 and 496,264 for faster RTs to the last-presented memory item, relative to other target-present probes. This indicates that the last-presented item was still in the focus of attention at all delays and thus, that no serial refreshing has occurred, even though the preprobe delays were considerably longer than in our previous studies.

**Table 1:** Evidence in the data for the last-presented benefit for each probe (Probe 2, 3, and 4) and for each preprobe delay (800, 1,100 or 1,500 ms) in Experiment 1

	Probe 2	Probe 3	Probe 4
800-ms delay	1623	496264	4298
1100-ms delay	48882	398461	18524
1500-ms delay	204	3202	12718

*Note.* Bayes factors are from paired, one-sided *t* tests testing the described benefit: faster responses for last-presented item compared with other target-present probes. The data of 30 participants was included in all tests reported in this table.

#### Discussion

The results of Experiment 1 indicate that the last-presented letter was still in the focus of attention, at all preprobe delays, and thus that no serial refreshing had occurred, even though all preprobe delay durations were now considerably longer than the time it may take to initiate refreshing. One could argue that, at least for Probe Positions 3 and Probe 4, responses are somewhat faster to probes matching the first memory item, relative to the mid-list positions, and that this may reflect some serial refreshing, at least of the first memory item. However, additional analyses (see Supplementary Materials) show that there is no evidence in the data of Experiment 1 for the notion that the serial position curves change meaningfully over time. An alternative account may be that participants were relying on verbal rehearsal to remember the red letters, thereby minimizing the need to engage in refreshing to maintain the information. To minimize the role of verbal rehearsal, Experiment 2 employed a spatial version of the probe-span task, presenting to-be-remembered locations in a random grid. The use of spatial stimuli in Experiment 2 also constitutes a first assessment of the evidence for serial refreshing beyond verbal working memory.

## Experiment 2

### Method

#### Participants

Thirty-nine undergraduate students at the University of Geneva participated and received partial course credit. All had normal or corrected-to-normal vision. In a first batch, 29 participants were tested. After that, testing continued until we reached 30 data sets after performance-based exclusions (see below). The sample size is based on similar studies using the probe-span task (e.g., Vergauwe et al., 2018).

#### Materials and procedure

A spatial version of the probe-span task was created by replacing letters by spatial locations. In particular, memory items were locations in a grid. We used a screen-wide grid) containing 16 randomly distributed squares (size 5 × 4 cm).^1^ On each trial, four of these squares were filled in red, representing the four to-be-remembered locations. The location of the red squares was chosen randomly without replacement from the 16 possible locations. Thus, no location was repeated within a series. The grid with to-be-remembered locations was shown screen-wide (with the center of the grid in the center of the screen), and each location was followed by a brief mask presented for 50 ms. The mask consisted of the same grid with all 16 locations presented in red (see Fig. 1b). Probes consisted of the same grid with a single location presented in black. At the end of the trial, locations were to be recalled by clicking on the corresponding unfilled locations shown on screen using the mouse, in order of presentation.

Overall, the task and procedure were the same as in Experiment 1, except for the stimuli and the following modifications. First, the presentation rate of the memory items and probes was slowed down. Memory items and probes were presented for 1,500 ms in Experiment 2. Second, two preprobe delay conditions were used instead of three; preprobe delay duration was either 150 ms or 1,200 ms. Third, regardless of the preprobe delay condition, the interval between two red locations was kept constant at 3,050 ms (50-ms mask, followed by 3,000-ms window during which the probe was presented). Depending on the experimental condition defined by preprobe delay, the delay between the offset of the mask and the onset of the black probe location was different (150 or 1,200 ms) and the remaining delay between the offset of the black location and the onset of the next red location differed as a function of preprobe delay as well (1,350 or 300 ms, respectively). Fourth, there were 96 experimental trials in total. Finally, to optimize data collection and ensure enough data points for each of the relevant experimental cells (i.e., last-presented vs. not-last-presented probes), we modified the distribution of probe identity relative to Experiment 1. In particular, at each probe position, the probe corresponded in approximately one third of the trials to a randomly new location, in approximately one third of the trials to the last-presented red location, in approximately one third of the trials to any of the to-be-remembered red locations presented up to that point except for the last one (in this last case, each red location presented up to that point in the trial, except the last-presented, had equal chances of being used as target-present probe). Importantly, in each of these four pools, every different probe type was associated approximately equally often with the two levels of preprobe delay. Like in Experiment 1, recall performance was scored with respect to serial order within each series (max = 4), and an average across all series was calculated per participant.

### Results and discussion

#### Performance-based exclusions

We applied the same performance-based exclusions as in Experiment 1 (which were based on Vergauwe et al., 2016, 2018). The data of one participant was discarded because of poor recall performance (i.e., less than one location out of four). All participants reached the 55% criterion of correct responses in the probe task, but the data of eight participants were discarded because they scored below 55% on the not-last-presented probes. These exclusions resulted in a final sample of 30 participants. These participants correctly recalled several memory items at the end of the series (*M* = 3.47, *SD* = .68) and had high accuracy on the probes (*M* = .95, *SD* = .07).

#### Last-presented benefit

**Fig. 3:**
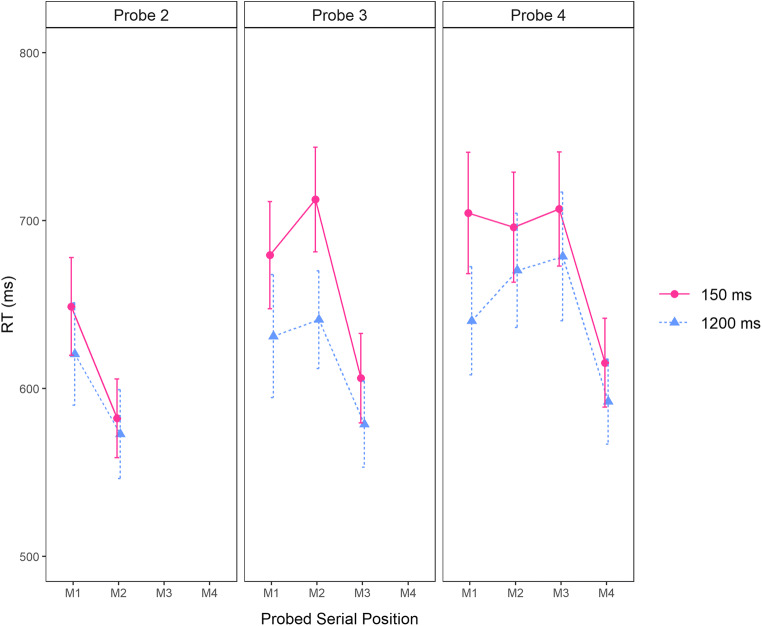
Mean probe response RT in ms observed in Experiment 2, as a function of the serial position of the matching memory item (probed serial position; on the *x*-axis) and probe position (Probe 2, Probe 3, or Probe 4 in the left, middle, and right panels, respectively). The delay following the probe appears as the graph parameter 150 or 1,200 ms). Error bars show standard errors of the mean

Serial position curves for the RTs of correct responses collected at Probe Position 2, 3, and 4 (i.e., the probe letters following Memory Items 2, 3, and 4, respectively) are shown in Fig. 3. As previously observed by Vergauwe et al. (2016, 2018), and in line with what we observed in our first experiment here, RTs were affected by the serial position of the matching memory item and became faster over time, but the serial position curves did not drastically change over time (i.e., with longer preprobe delay durations). Importantly, the last-presented item was the fastest responded to, again at all probe positions and for all preprobe delay durations.

We tested the serial refreshing hypothesis again by examining the evidence for or against a last-presented benefit at each preprobe delay duration. For each preprobe delay duration, and for each probe position, a separate one-sided Bayesian *t* test was run (i.e., a total of 6 t tests), testing whether the RTs to probes matching the last-presented memory item were faster than RTs to other target-present probes. Only correct responses were included. As can be seen in Table 2, the evidence for a last-presented benefit was again very to extremely strong at all preprobe delay durations, with Bayes factors ranging between 22.38 and 73,844 for a last-presented RT benefit. Thus, like with verbal memory materials, faster responses were observed for the last-presented memory item using visuospatial materials, indicating that the last-presented location was still in the focus of attention at all delays and thus, that no serial refreshing had occurred in visuospatial working memory.

**Table 2:** Evidence in the data for the last-presented benefit for each probe (Probe 2, 3, and 4) and for each preprobe delay (150 or 1,200 ms) in Experiment 2

	Probe 2	Probe 3	Probe 4
150 ms delay	192	73844	545
1200 ms delay	22.38	37.29	1354

*Note.* Bayes factors are from paired, one-sided *t* tests testing the described benefit: faster responses for last-presented item, compared with other target-present probes. The data of 30 participants was included in all tests reported in this table.

#### Discussion

The results of Experiment 2 indicate that the last-presented memory item remained in the focus of attention, at all preprobe delays. This indicates that no serial refreshing had occurred, even though we used spatial locations as memoranda. Experiment 2 used randomized spatial locations to minimize the involvement of verbal rehearsal. Although we cannot rule out *any* involvement of verbal rehearsal in our spatial task, we believe it is reasonable to assume that replacing letters by randomized locations results in a drastic reduction of the spontaneous use of verbal rehearsal, based on existing research (discussed in the introduction). Doing so, we still did not observe evidence for serial refreshing. Thus, like in verbal working memory, we found no evidence for serial refreshing in visuospatial working memory.

## General discussion

The current study follows up on previous findings of Vergauwe et al. (2016, 2018), who consistently failed to find evidence for serial refreshing in two series of experiments using verbal probe-span tasks. Here, we proposed and tested two alternative accounts. According to the first account, previous studies did not find evidence for serial refreshing because the inclusion of very short preprobe delay durations (i.e., preprobe durations that are shorter than the time it takes to initiate refreshing) created a task context that discourages participants to engage in refreshing. To examine this possibility, the current Experiment 1 used a verbal probe-span task in which the longest preprobe duration from previous studies (i.e., 800 ms) was embedded in a task context that only includes preprobe durations that are considerably longer than the delays that were used in the previous studies. Importantly, all preprobe delays were now longer than 400 ms and thus, longer than the time it takes to initiate refreshing (M. R. Johnson et al., 2015). Based on the assumption that participants are faster to respond to the item that is currently in the focus of attention, we expected that the RT benefit for the last-presented item would disappear at longer preprobe delays if serial refreshing occurs spontaneously between the memory items and the probes. However, despite the use of longer preprobe delays and thus creating a slower task context, the findings of the current Experiment 1 contrast sharply with this prediction; RTs to the last-presented item were the fastest at all preprobe delays, suggesting that the last-presented item remained in the focus of attention and, thus, that no serial refreshing had occurred between the memory items and the probes. As such, together with the previous studies of Vergauwe et al. (2016, 2018), we have failed to find evidence for spontaneous serial refreshing in verbal working memory across seven experiments using the probe-span task with verbal memory materials.

One reason for this consistent lack of evidence for serial refreshing may be the consistent use of verbal memory materials in the previous experiments. Verbal memory materials are known to have more than one maintenance mechanism at their disposal (see Camos, 2017, for a recent review). In particular, the letters that were used in the previous experiments can easily be rehearsed and, therefore, it is possible that no evidence for serial refreshing was found in the previous experiments. Indeed, even though several experiments used phonologically similar letters as memoranda, for which Camos et al. (2011) have shown that participants favor refreshing over rehearsal, one could still argue that participants used verbal rehearsal to a certain level, thereby preventing the detection of serial refreshing. In contrast to the previous experiments, the current Experiment 2 employed a spatial version of the probe-span task. Moreover, we used locations in a random grid as memory materials to further minimize the use of verbal encoding. Even though random spatial locations were used in Experiment 2, very strong evidence was found for a last-presented benefit, both at short and long preprobe delays. This indicates that the last-presented item was still in the focus of attention when the probe was presented and thus, that no serial refreshing had occurred between the memory items and the probes in the spatial probe-span task. As such, we failed to find evidence for the spontaneous use of serial refreshing in visuospatial working memory. Thus, together with the previous studies, across a set of eight experiments in total, a clear and coherent pattern of invariance emerges, whereby a last-presented benefit is observed at all preprobe delays, for both verbal and visuospatial memory materials. Below, we discuss three different accounts of this pattern, together with the corresponding theoretical implications.

According to the first account of the observed pattern, our findings establish the existence of clear boundary conditions to the spontaneous occurrence of serial refreshing. Indeed, in none of the probe-span tasks did we observe evidence for serial refreshing. However, other studies did find evidence for spontaneous serial refreshing using the disappearance of the last-presented benefit as index for the occurrence of serial refreshing (Valentini et al., 2022; Vergauwe & Langerock, 2017). These studies used an item recognition task, rather than probe-span task. In these tasks, short lists of letters were presented, followed by a single probe letter which needed to be judged present in or absent from the list. Very strong evidence was observed for a last-presented benefit when the probe was presented immediately after fast list presentation. However, when time for refreshing was provided, either by slowing down the rate at which the memory lists were presented or by inserting an empty delay between the last-presented item and the probe, the last-presented benefit disappeared, indicating that serial refreshing had occurred during (Vergauwe & Langerock, 2017) or after list presentation (Valentini et al., 2022; Vergauwe & Langerock, 2017), respectively. Therefore, it appears that the spontaneous use of serial refreshing is task dependent. In particular, some task contexts may encourage the use of serial refreshing, whereas other task contexts discourage its use (see also Vergauwe et al., 2019, for a similar argument). This could explain why evidence for serial refreshing can be found in some task contexts, but not in others. Assuming that task characteristics matter for the spontaneous occurrence of serial refreshing implies that serial refreshing is less generally used than previously thought. In particular, inconsistent spontaneous use of serial refreshing makes it unlikely that serial refreshing plays a major role in maintaining information over brief periods of time. That is not to say that serial refreshing could not play a role in working memory, but its role may be much more limited than previously thought.

According to the second account, our findings provide evidence against the assumed serial nature of refreshing but could be accounted for by proposing other refreshing schedules. Indeed, spontaneous refreshing is typically assumed to occur in a serial, forward-cumulative fashion (e.g., Barrouillet & Camos, 2012; Cowan, 2011; McCabe, 2008; Nee & Jonides, 2013; Vergauwe et al., 2014; Vergauwe & Cowan, 2014), whereby refreshing starts with the first-presented memory item, and follows the order of presentation (e.g., refreshing the list K-B-N-S, starting from the first item and in the same order, thus as “K-B-N-S”). There is, however, currently not much evidence for this notion, and the findings of a recent study were inconsistent with the idea that cumulative, forward-order refreshing of a letter list results in better memory performance (relative to refreshing the list in a random order, e.g., refreshing the list K-B-N-S as follows: “N-S-B-K”; Vergauwe, 2018). However, even if we assume that serial refreshing occurs in an order that is not cumulative and forward-ordered, the interpretation of our results would not change drastically. For example, one alternative refreshing schedule that has been proposed is refreshing of the least-activated information first (e.g., Lemaire et al., 2018; Oberauer & Lewandowsky, 2011; Portrat & Lemaire, 2015). This least-activated-first refreshing account would make similar predictions as we made here, with the focus of attention switching away from the least-presented memory item as soon as refreshing is occurring. Indeed, the last-presented item is highly unlikely to be the last-activated memory item in working memory right after its presentation and thus, as soon as refreshing would start, the focus of attention would switch to another memory item and thus, the last-presented benefit would disappear as soon as refreshing occurs. Therefore, we think that our interpretation of the current findings does not depend on how serial refreshing is implemented, as long as refreshing is not considered as operating only on the last-presented memory item. It is worth noting that, if refreshing would be considered as operating on the last-presented memory item only, then it would become indissociable from consolidation (see Ricker et al., 2018, for a recent review) and no longer be considered as a list-wide, serial maintenance mechanism, which would be drastically different from common conceptions of refreshing (see Camos et al., 2018, for a recent review).

The first two accounts worked under the assumption that the last-presented benefit reflects the increased accessibility of the last-presented item and the disappearance of this benefit reflects the occurrence of serial refreshing. However, according to a third account, the last-presented benefit does not reflect the presence of the last-presented item in the focus of attention. One could, for example, assume that the last-presented benefit is simply reflecting the unequal distribution of memory strength (or activation levels) across serial positions of a list (e.g., Donkin & Nosofsky, 2012; Monsell, 1978; Niklaus et al., 2019). Factors such as temporal distinctiveness and retroactive representational interference could, potentially together with other variables, lead to a certain pattern of strength across the different items represented in working memory (see Niklaus et al., 2019, for a similar argument). Often, these factors would lead to a particularly high level of activation for the last-presented memory item, resulting in the presence of a last-presented benefit in many task situations. Some changes in the pattern of memory strength across list items may then result in the disappearance of the last-presented benefit, while others do not. This could account for the fact that the disappearance of the last-presented benefit is observed in some task situations, but not in others. However, we think that, even under this assumption, it is reasonable to expect the disappearance of the last-presented benefit over time, if serial refreshing occurs. Indeed, refreshing is assumed to reactivate and boost memory representations (see Camos et al., 2018), and thus changes in the pattern of activation levels across list items are to be expected if serial refreshing occurs. However, one could argue that parallel refreshing can occur during preprobe delays without changing the pattern of activation levels across list items and thus, without disappearance of the last-presented benefit. Therefore, we must conclude that our findings go against any implementation of serial refreshing of verbal and visuospatial materials, but not against implementations of refreshing that operate nonserially.

To conclude, the current results extend the previous findings of Vergauwe et al. (2016, 2018) and provide further evidence against the prominent hypothesis of serial refreshing in both verbal and visuospatial working memory, at least in the probe-span task. Taken together, these studies strongly suggest that either (1) serial refreshing does not occur spontaneously in all situations requiring the maintenance of information over brief periods of time, (2) refreshing does not operate serially, or (3) refreshing does not occur spontaneously and an alternative explanation exists for the disappearance of the last-presented benefit in those situations in which it has been observed in previous studies.

## Supplementary Information

ESM 1 (DOCX 306 kb)

## Data Availability

All data are available on Open Science Framework: https://osf.io/5w6zd/ (10.17605/OSF.IO/5W6ZD)

## Footnotes

1 A set of 16 locations was chosen, to stay close to what is often used in the working memory literature. Indeed, often, 4 × 4 matrices are used, resulting in 16 locations. To minimize verbal recoding, we presented the locations in a random grid instead of in matrices.
