# Supplementary material for: A (further) test of spontaneous serial refreshing in verbal and spatial working memory
Source: Atten Percept Psychophys. 2022 Dec 1;85(5):1600–11. doi: 10.3758/s13414-022-02624-x (PMC10371951; doi:10.3758/s13414-022-02624-x)
Supplement: Supplementary file 1 — (DOCX 306 kb) [file 13414_2022_2624_MOESM1_ESM.docx]

**Supplementary materials**

As can be seen in Figure 2, responses appear to be faster to probes matching the first memory item, compared to mid-sequence positions, for probe positions 3 and 4. At the same time, responses to probes matching the first memory item also appear consistently slower than responses to probes matching the last-presented memory item. As suggested by an anonymous reviewer, we explored the difference in RTs to probes matching the first-presented vs. the last-presented item in more detail. First, we ran a series of one-sided t-tests assessing the evidence in the data for responses being faster for probes matching the last-presented memory item, compared to probes matching the first-presented memory item, for each probe (Probe 2, 3 and 4) and for each pre-probe delay (800, 1100 or 1500 ms) in Experiment 1. As can be seen in the table below, these t-tests confirmed that responses to the last-presented memory item were consistently faster than responses to the first-presented memory item, at all probe positions and all pre-probe delays (all BFs > 10). Additionally, we ran a BANOVA at each probe position, with the within-subject variables Delay (800 ms, 1100 ms, and 1500 ms) and Serial position of the matching memory item (first-presented vs. last-presented memory item). If the first-presented memory item is more subject to refreshing, then one could expect an interaction between Delay and Serial position, with longer Delays resulting in smaller differences between RTs to probes matching the first-presented vs. last-presented memory item. Instead, the BANOVAs showed that the best model of the data collected at each probe position is the main effects-only model, including the main effects of Delay and Serial position of the matching memory item, but not their interaction (BF’s against full model including also the interaction: 1.45, 6.81, and 7.11, for Probe positions 2, 3, and 4, respectively). Repeating these BANOVAs for probe positions 3 and 4, this time considering all target-present probe types (i.e., memory item 1, 2, or 3, for Probe position 3; and memory item 1, 2, 3, or 4, for Probe position 4) confirmed this pattern; the best model was the model with only the two main effects of Delay and Serial position of the matching memory item (BF’s against full model including also the interaction: 27.29 and 40.22, for Probe positions 3 and 4, respectively). Thus, across the three probe positions, there is no evidence in the data of Experiment 1 for the notion that the serial position curves change meaningfully over time.

**Table**

*Evidence in the data for responses being faster for probes matching the last-presented memory item, compared to probes matching the first-presented memory item, for each probe (Probe 2, 3 and 4) and for each pre-probe delay (800, 1100 or 1500 ms) in Experiment 1. Bayes factors are from paired, one-sided t-tests testing the described effect. The data of 30 participants were included in all tests reported in this table, except for the test at Probe position 4 - 800 ms Delay, where the data of only 29 participants were included because one participant did not have correct responses to probes matching memory item 1 at Probe position 4 with Delay 800 ms.*

|  | **Probe 2** | **Probe 3** | **Probe 4** |
| --- | --- | --- | --- |
| **800 ms delay** | 1624 | 1783 | 36.50 |
| **1100 ms delay** | 48882 | 5214 | 159 |
| **1500 ms delay** | 204 | 60.58 | 10.32 |

**Figure 1**

**A**

**
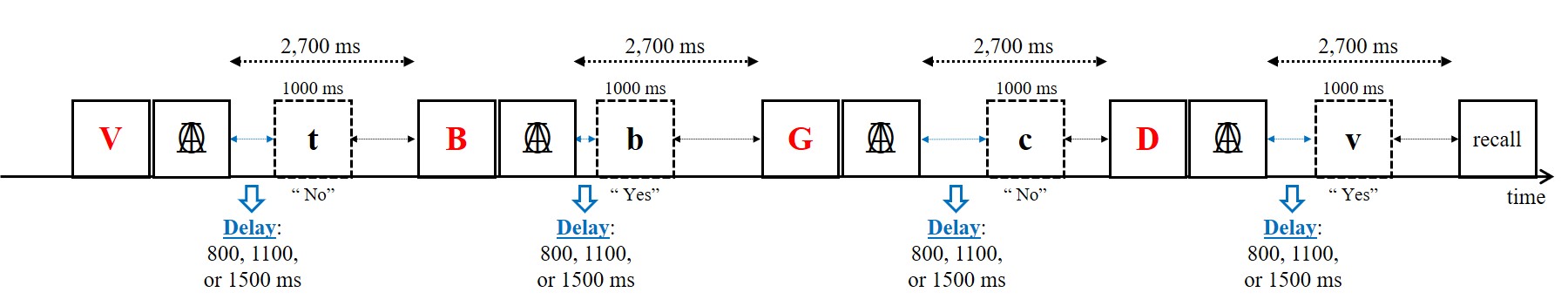
**

**B**

**
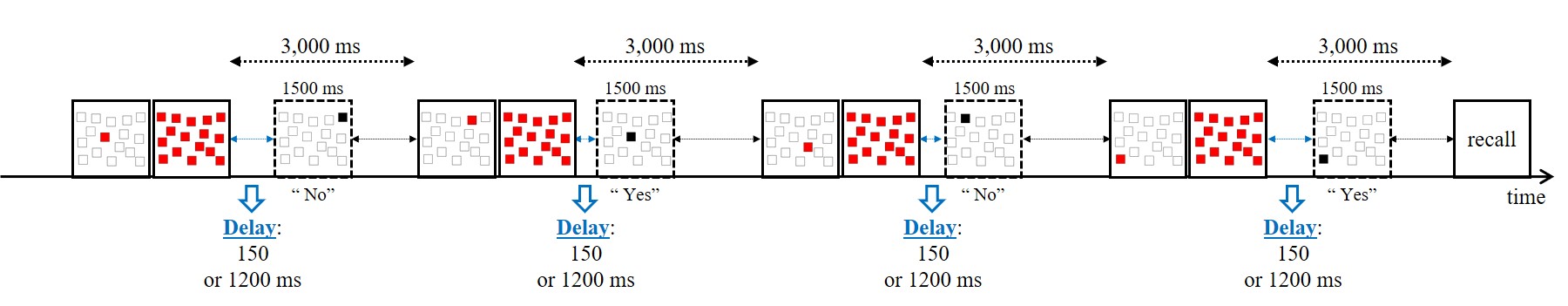
**

Illustration of a trial within the probe-span task used in Experiment 1 (Panel A) and in Experiment 2 (Panel B). Series of four red memory items were presented (and masked) for subsequent recall and black probe items were presented between the items to be remembered, with each probe to be judged present in or absent from the list presented so far. At the end of the series, participants recall the four memory items in order of appearance. The delay before the probe was manipulated (800, 1100, or 1500 ms in Experiment 1; 150 or 1200 ms in Experiment 2).

**Figure 2**


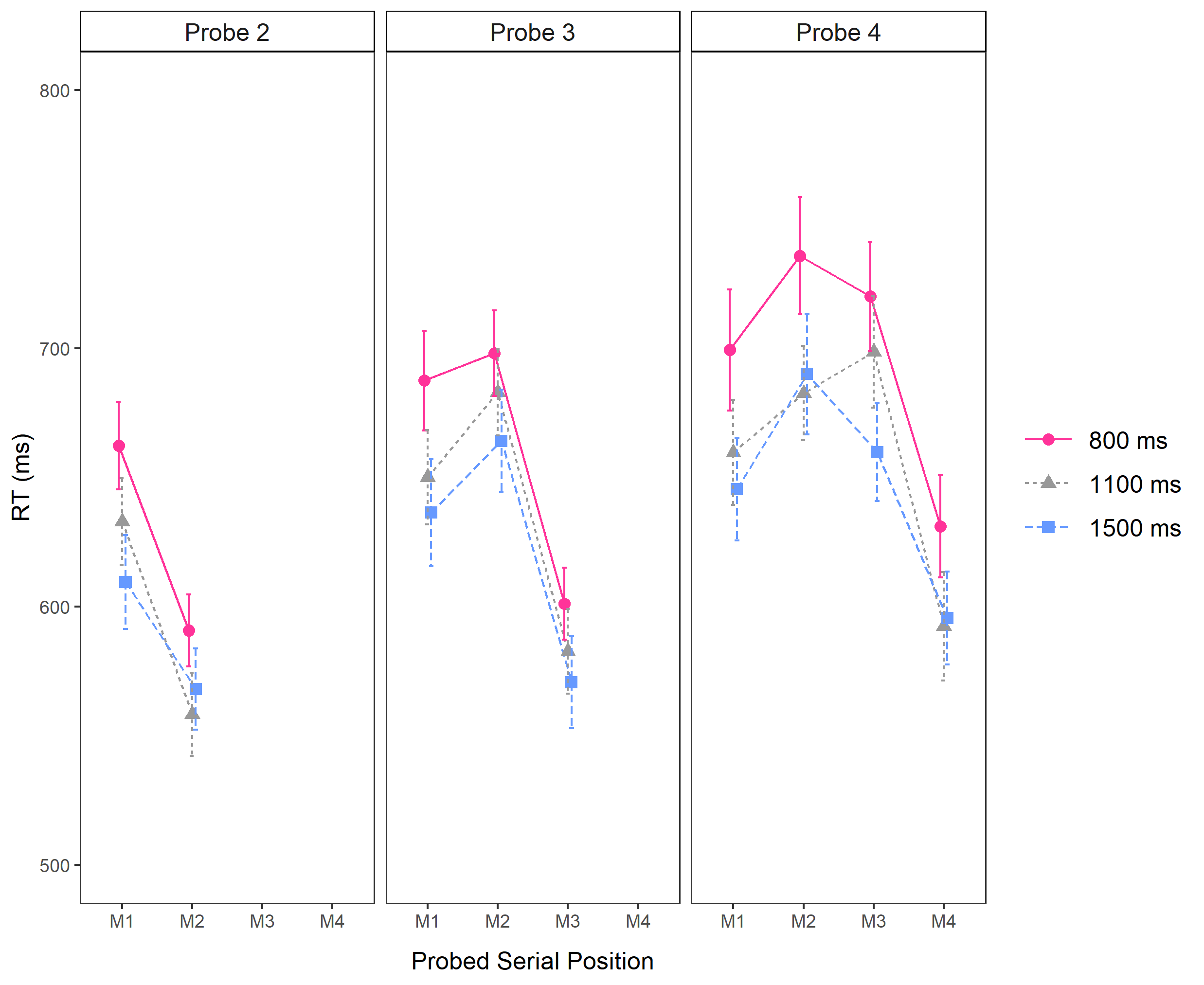


Mean probe response RT in ms observed in Experiment 1, as a function of the serial position of the matching memory item (Probed serial position; on the x axis) and probe position (Probe 2, Probe 3, or Probe 4 in the left, middle and right panels, respectively). The delay following the probe appears as the graph parameter 800, 1100 or 1500 ms). Error bars show standard errors of the mean.

**Figure 3**


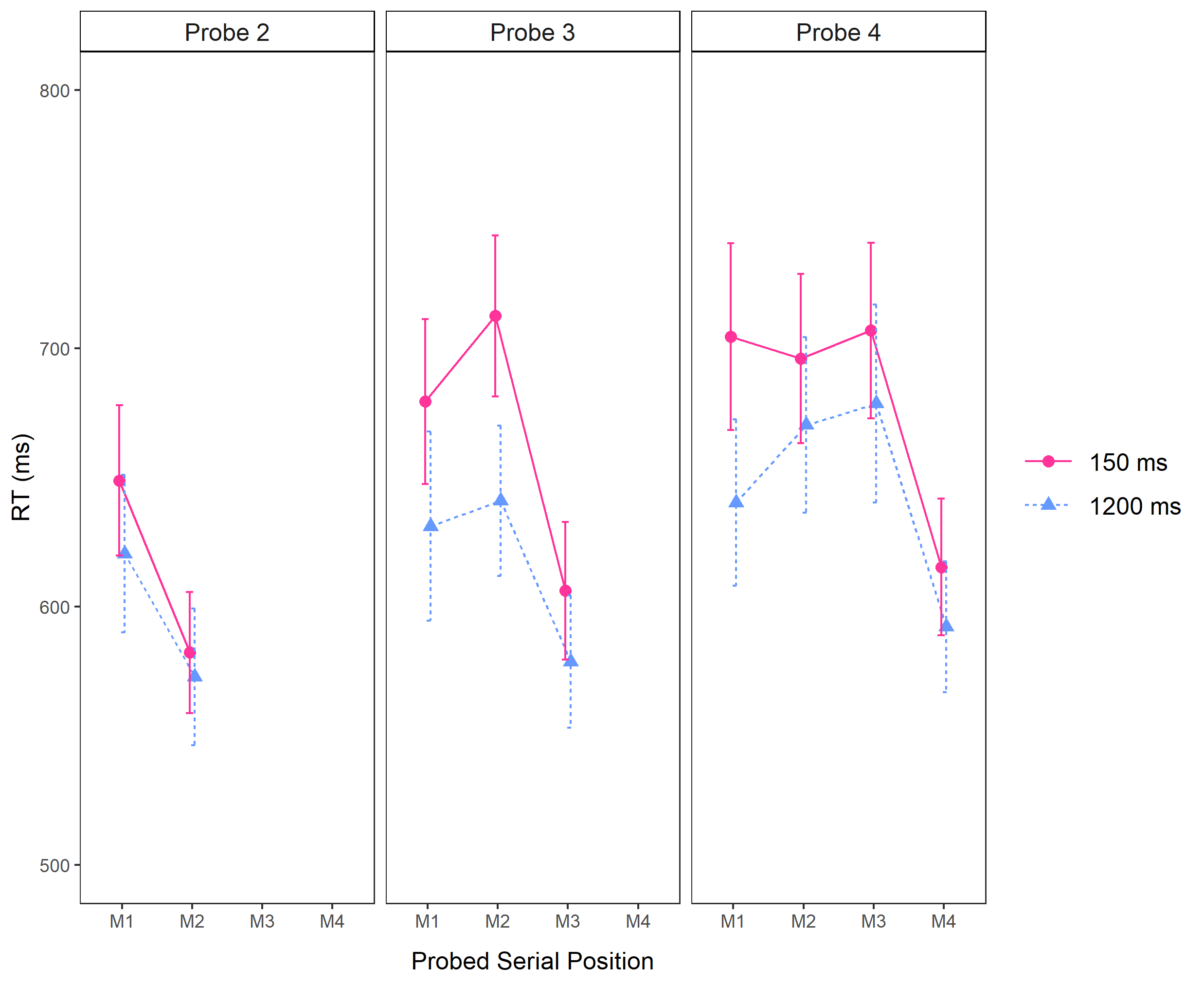


Mean probe response RT in ms observed in Experiment 2, as a function of the serial position of the matching memory item (Probed serial position; on the x axis) and probe position (Probe 2, Probe 3, or Probe 4 in the left, middle and right panels, respectively). The delay following the probe appears as the graph parameter 150 or 1200 ms). Error bars show standard errors of the mean.
